# The desmosome comes into focus

**DOI:** 10.1083/jcb.202404120

**Published:** 2024-08-09

**Authors:** Navaneetha Krishnan Bharathan, Alexa L. Mattheyses, Andrew P. Kowalczyk

**Affiliations:** 1 Departments of Dermatology and Cellular and Molecular Physiology, Pennsylvania State University, College of Medicine, Hershey, PA, USA; 2 Department of Cell, Developmental, and Integrative Biology, The University of Alabama at Birmingham, Birmingham, AL, USA

## Abstract

The desmosome is a cell–cell adhesive junction that provides integrity and mechanical resistance to tissues through its attachment to the intermediate filament cytoskeleton. Defects in desmosomes cause diseases impacting the heart, epidermis, and other epithelia. In this review, we provide a historical perspective on the discovery of the desmosome and how the evolution of cellular imaging technologies revealed insights into desmosome structure and function. We also discuss recent findings using contemporary imaging approaches that have informed the molecular order, three-dimensional architecture, and associations of desmosomes with organelles such as the endoplasmic reticulum. Finally, we provide an updated model of desmosome molecular organization and speculate upon novel functions of this cell junction as a signaling center for sensing mechanical and other forms of cell stress.

## Teaser

Bharathan and colleagues provide a historical perspective on the discovery of the desmosome and how imaging technologies have advanced our understanding of this adhesive intercellular junction.

## Introduction

**Figure 1.**
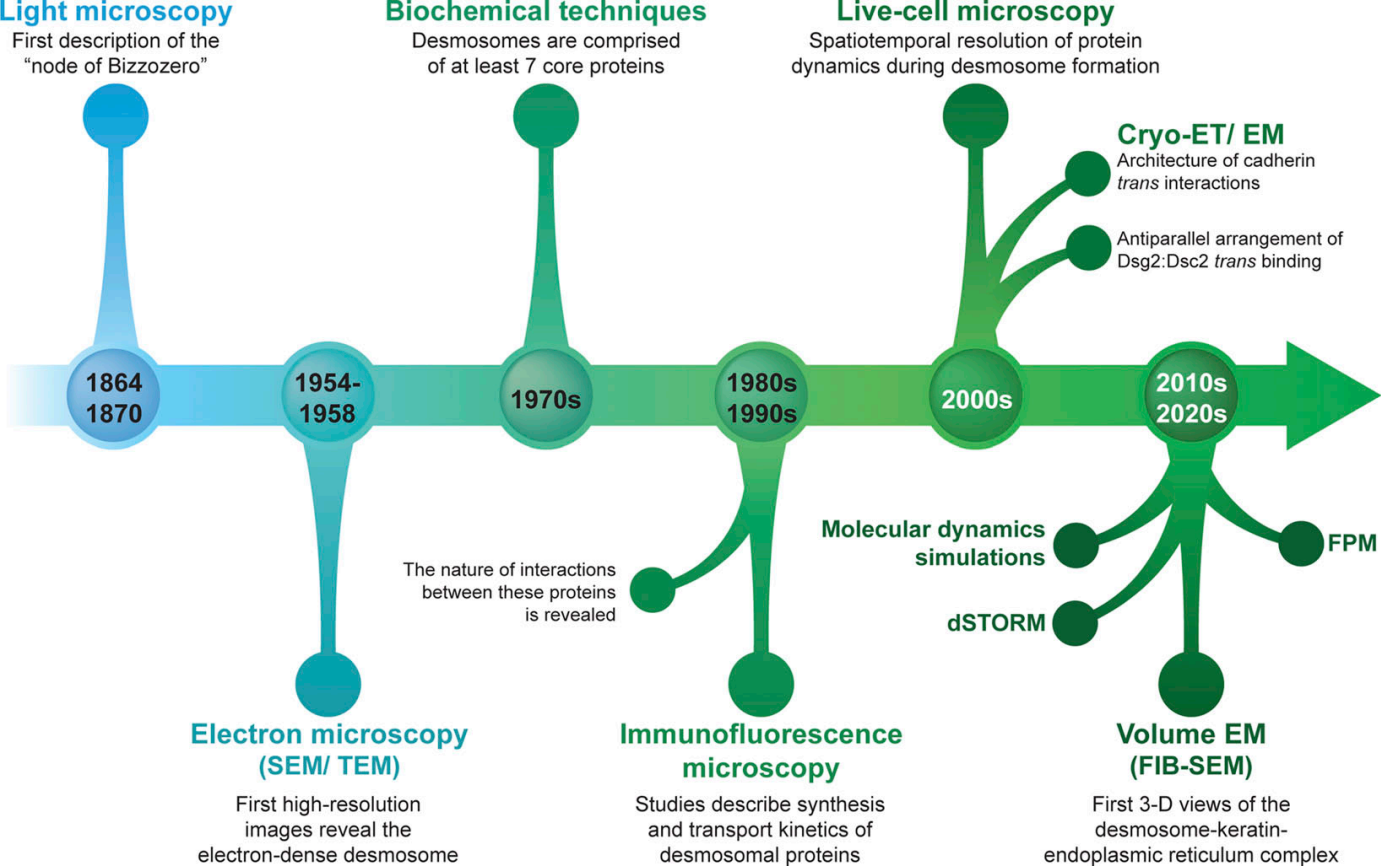
**Key findings in desmosome biology through the ages.** A timeline highlighting major discoveries in the field of desmosome biology at various points in history and the techniques that facilitated these insights.

The desmosome first arose evolutionarily in organisms such as jawless fish and other non-mammalian vertebrates (Green et al., 2020). Although evolutionarily emerging after the adherens junction, desmosomes were the first cell–cell junction to be visualized by microscopy more than 150 years ago (Bizzozero, 1864). Since their discovery, desmosomes have been linked to inherited, autoimmune, and infectious diseases, with desmosome dysfunction leading to skin, cardiac, and hair follicle defects (Celentano and Cirillo, 2017; Brooke et al., 2012; Stahley and Kowalczyk, 2015). Desmosome structure is conserved across vertebrate species, tissues, and cell culture models (Holbrook and Odland, 1975; Goonesinghe et al., 2012; Fleming et al., 1991; Garrod and Chidgey, 2008). For both early and modern microscopists, desmosome density and size make these junctions highly amenable to electron microscopy (EM) and optical imaging approaches. Here, we review the cell imaging techniques that have yielded deep insights and new views into the organization and function of this unique and essential adhesive intercellular junction (Fig. 1).

Early pioneers in anatomy and microscopy first visualized structures that appeared to connect the cytoplasm of adjacent epithelial cells in the skin. These structures were initially called “cellular bridges,” attributed to predictions by Theodor Schwann (Schwann, 1839), who suggested that animal cells might fuse their cytoplasm through these connections. Otto von Schrön presented his investigations of thin sections of the Malpighian or spinous layer (*stratum spinosum*) of the epidermis, reporting the presence of eponymous “spines” between adjacent cells, as well as an intercellular cementing substance (Schrön, 1863). He described that the spines, later identified as the desmosome–keratin complex, were surrounded by a membrane. He further posited, incorrectly as it turned out, that spines of adjacent cells were connected to each other through “pores” in the membrane. Independently, Max Schultze (Schultze, 1864), using an “iodine serum” fixation technique on ox, sheep, and human tongue epithelia, challenged the notion of connected cytoplasms, instead reporting that these spines between adjacent cells “interlocked” like the bristles of two toothbrushes in contact.

**Figure 2.**
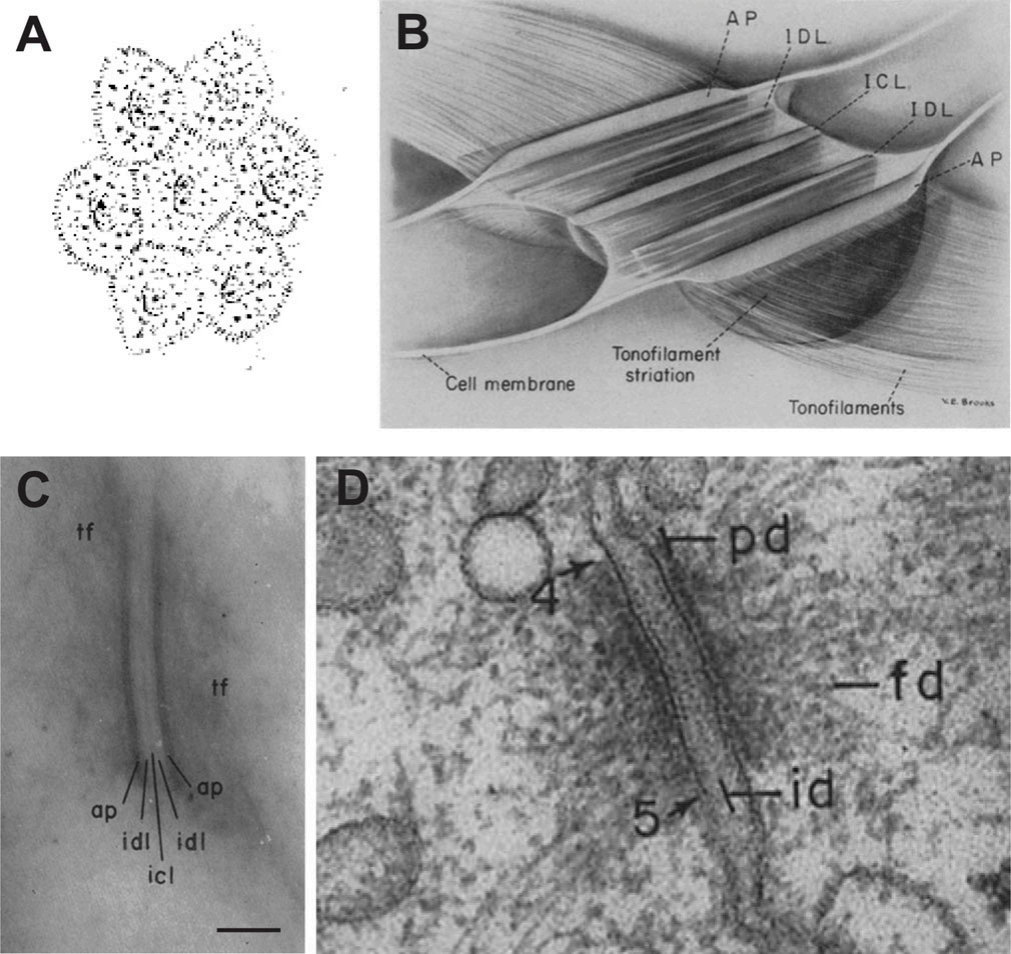
**Visualizing and illustrating desmosome ultrastructure. (A)** Bizzozero’s drawings of a group of vulvar epithelial cells (Bizzozero, 1864). Note the spines at the cell–cell contacts. **(B)** Schematic illustration of the ovoid desmosome as conceived by George Odland from his studies in human skin. The various layers of the desmosomal plaque are specified. Reproduced/adapted from Odland (1958). **(C)** Electron micrograph of the desmosome cell–cell junction in human skin depicting the attachment plaque (AP/ap), the intercellular contact layer (ICL/icl), the intermediate dense layers (IDL/idl), and tonofilaments (tf). Scale bar = 100 nm. Reproduced/adapted from Odland (1958). **(D)** Electron micrograph of a desmosome in the epithelium of rat intestinal mucosa. The arrows “4” and “5” point to the edges of the desmosome. Notice the keratin filament bundles or cytoplasmic fibrils (fd) terminating at the dense plate (pd) on either side of the desmosome. The intermediate line (id) bisecting the desmosome is the same structure as the icl in C. Reproduced/adapted from Farquhar and Palade (1963).

In 1864, Guilio Bizzozero published findings that correctly interpreted these spines in adjacent cells as being separated by their respective cell membranes (Fig. 2 A) (Bizzozero, 1864). In 1870, using better microscopy techniques, Bizzozero saw that spines from adjacent cells formed end-to-end contact (Bizzozero, 1870). He also reported a swelling, or “node,” at the interface of apposed spines, which was coined the node of Bizzozero. These nodes of Bizzozero, sometimes also referred to as “knots of Ranvier” (Ranvier, 1879, 1882), eventually came to be known as “desmosomes,” a term coined much later by Josef Schaffer (Schaffer, 1920). Desmosome is derived from the Greek words “desmos,” meaning bond or fastening, and “soma,” meaning body.

## EM reveals the ultrastructure of the desmosome

The invention of the electron microscope in 1931 by Ernst Ruska and Max Knoll heralded a new age in cell biology (Knoll and Ruska, 1932) by allowing scientists to observe life at unprecedented magnification and resolution. George E. Palade pioneered the use of a buffered osmium tetroxide fixative, which preserved structures better than acidic formaldehyde-based fixatives (Palade, 1952) and provided contrast to cellular structures for EM imaging. The first transmission EM (TEM) studies of normal human skin were performed using variations of this osmium fixative and celloidin or paraffin embedding to “harden” the tissues (Adolph et al., 1951; Pease, 1951; Gray et al., 1952). Concurrent advances in microtome sectioning capabilities allowed researchers to achieve very thin sections of 0.1–0.2 µm. Thin sections permitted greater penetration of the electron beam and provided higher-resolution images of electron-dense cytoskeletal filaments. These studies also reported electron-dense “nodes” or “granules” at the interface of two adjacent cells, although the term desmosome did not see widespread use to describe these nodes at the time. Despite the many technical advances, fixation artifacts made these structures hard to resolve, and regions of cell–cell contact still appeared to have contiguous membranes.

Keith Porter was the first to describe the fine structure of desmosomes. He made his observations in the epidermis of *Ambystoma* (salamander) larvae, reporting that apposing filamentous bundles terminated at a layer of dense material subjacent to the cell membrane (Porter, 1954). He noted that these bundles did not cross from one cell to another. In 1955, Cecily C. Selby published an EM study of the epidermis of various mammals (Selby, 1955). Importantly, she employed an “electron stain” of phosphotungstic acid to increase the contrast of electron-dense structures. With this technique, Selby was able to better visualize the fine structure of keratin filaments, which she termed “tonofilaments.” In a follow-up study of the layers of human skin, Selby observed that tonofilaments terminated at desmosome halves on either side of a cell–cell contact and were not continuous across the intercellular space, confirming Porter’s findings in the salamander (Selby, 1957). Desmosomes appeared as electron-dense plaques, one on either side of the cell–cell junction, with an intermembrane gap of ∼300 Å.

In 1958, George Odland published a comprehensive study of desmosome ultrastructure, unveiling its complexity. In human skin samples cut tangential to the plane of the plaque, he revealed the ovoid or disc-shaped nature of the desmosome. He also presented a more highly resolved electron density pattern of the desmosome at the cell–cell contact compared with observations made by Porter and Selby (Odland, 1958). In transverse sections of human skin, Odland observed that desmosomes had five dense stripes, with a thickness of ∼500 Å and a diameter of 0.3–0.7 µm (Fig. 2, B and C). The most intracellular stripe or “attachment plaque” was the region at which keratin filaments terminated. This band is now called the inner dense plaque (IDP). The IDP was followed by a thinner stripe called the intermediate dense layer, now known as the outer dense plaque (ODP), subjacent to the plasma membrane. Thus, the desmosome is symmetrical and comprises an IDP and ODP in each cell, appearing as mirror images on either side of the cell–cell contact. At the center of this pair of plaques lies a thin, dense lamina called the intercellular contact layer, now called the desmosome midline or central dense stratum. Later EM studies described similar desmosome ultrastructure in other tissues and species, including frog mesothelium and the stomach, jejunum, and colon epithelia of rats and guinea pigs (Fig. 2 D) (Hama, 1960; Farquhar and Palade, 1963). Regions of electron density with similar patterning are also observed in the intercalated disc of the cardiac muscle, known to contain elements of both adherens junctions and desmosomes (Sjöstrand and Andersson, 1954; Muir, 1957; Franke et al., 2006).

Freeze-fracture EM proved a convenient method to obtain en face views of desmosome extracellular surfaces. This technique involves using a vacuum to break or fracture a frozen sample followed by the creation of a platinum replica of the exposed surfaces. Samples fracture along the plane of least resistance, such as the hydrophobic zone within a lipid bilayer, exposing surfaces of membrane proteins. Desmosomes in freeze-fracture electron micrographs appeared as regions of densely packed granular particles (presumably the desmosomal cadherins), differentiating them from the surrounding desmosome-free membrane (Breathnach, 1973; Breathnach et al., 1973; Kelly and Shienvold, 1976; Arnn and Staehelin, 1981).

The unprecedented resolution achieved in these early EM studies gave rise to the idea that desmosomes were adhesive structures mediating the attachment of adjacent cells within a tissue. Testing this idea meant deciphering the protein composition of the desmosome and the spatial organization, or architecture, of its components.

## Biochemical characterization of the desmosome

Pioneers such as Christian de Duve, George Palade, Albert Claude, and others combined biochemical approaches with EM to establish the foundation of “cell biology” as a discipline. These approaches were applied to the desmosome in the 1970s and 1980s to reveal the proteins that comprise the electron-dense ultrastructure viewed in tissues and cells and allowed for antibody production and, ultimately, cDNA cloning of desmosome components.

Biochemical characterization began with isolation of intact desmosomes from bovine snout (Skerrow and Matoltsy, 1974b, 1974a; Drochmans et al., 1978). Skerrow and Matoltsy pioneered a tissue homogenization and sucrose gradient fractionation workflow, followed by EM of the various sucrose fractions. This protocol revealed Fraction III (at the 55–60% sucrose interface) as the desmosome-containing fraction (Skerrow and Matoltsy, 1974b). Isolated desmosomes contained proteins, carbohydrates, and lipids in a 76:17:10 ratio (Skerrow and Matoltsy, 1974a). The lipid fraction was made up of ∼40% cholesterol and 60% phospholipid. Using ion exchange chromatography, they determined the total amino acid composition of the desmosome fraction, noting a relatively high proportion of nonpolar residues. Electrophoresis with SDS-PAGE revealed 24 protein bands ranging from 15 to 230 kDa, where seven bands of the highest intensity made up ∼81% of the preparation. Subsequent periodic acid-Schiff treatment also revealed that two of the seven bands (Bands 3 and 4) were likely glycoproteins, which we now know include the desmosomal cadherins desmoglein (Dsg) and desmocollin (Dsc) (Cowin et al., 1984; Buxton et al., 1993; Gorbsky and Steinberg, 1981). Skerrow and Matoltsy characterized the five remaining major bands (Bands 1, 2, 5, 6, and 7) on the basis of size only, suggesting that they made up the desmosomal plaque. Although the remaining 17 bands were not further examined, it is possible that they correspond to the smaller keratin isoforms, corneodesmin, Perp, and other proteins (Serre et al., 1991; Simon et al., 1997; Garrod and Chidgey, 2008; Ihrie et al., 2005; Badu-Nkansah and Lechler, 2020; Hegazy et al., 2022).

The identity, dynamics, localization, and function of the seven core desmosomal proteins were the main focus of the next desmosome imaging revolution. For more details, we refer readers to a number of historical and contemporary reviews that discuss the biochemical composition and protein interactions of desmosome constituents (Holthöfer et al., 2007; Delva et al., 2009; Green and Simpson, 2007; Garrod and Chidgey, 2008; Harmon and Green, 2013). Briefly, Dsg and Dsc are transmembrane adhesion molecules and are members of the cadherin gene superfamily (Buxton et al., 1993). These desmosomal cadherins engage in cis and trans interactions to form highly clustered complexes that mediate strong cell–cell adhesion. The cytoplasmic tails of the desmosomal cadherins bind to plakoglobin (PG), an armadillo family protein related to β-catenin (Peifer et al., 1992). In turn, plakoglobin interacts with desmoplakin (DP), a member of the plakin family (Green et al., 1992; Kowalczyk et al., 1997). DP binds to the keratin intermediate filament (IF) cytoskeleton, thereby integrating the cadherin adhesive interface with the cytoskeleton. Plakophilins (PKP), also members of the armadillo family (Hatzfeld et al., 1994), interact with DP, PG, Dsg1/2, and Dsc1a/2a (Smith and Fuchs, 1998; Chen et al., 2002; Kowalczyk et al., 1999; Hatzfeld et al., 2000). PKPs promote desmosome formation through the recruitment and clustering of the desmosomal cadherin complexes at the plasma membrane (Bornslaeger et al., 2001; Wahl, 2005; Hatzfeld, 2007), facilitating strong adhesion and keratin linkages. Importantly, early biochemical and gene sequencing studies formed the foundation for the development of antibody and cDNA reagents that led to a revolution in desmosome studies that would build on early EM analysis of desmosome morphology.

## A new view: Desmosomes as a dynamic cell–cell junction

After the initial identification of desmosome structure and protein composition, considerable attention turned to understanding desmosome regulation. One of the first studies of desmosome assembly was conducted in the developing chick blastoderm using EM (Overton, 1962). Overton described three stages of desmosome formation, with the initial appearance of an electron-dense region at the cell–cell interface, followed by condensation of the desmosomal plaque, and finally the appearance of the keratin filaments at the plaque. Subsequent studies showed that desmosome formation in epithelial cells can be induced by the elevation of extracellular calcium concentration from <0.1 mM to more physiological levels of ∼1.8–2 mM (Hennings and Holbrook, 1983; Hennings et al., 1980; Watt et al., 1984; Mattey and Garrod, 1986a). This calcium switch protocol, in combination with indirect immunofluorescence microscopy using antibodies raised against desmosomal proteins, facilitated the investigation of desmosome assembly in fixed cells (Watt et al., 1984; Pasdar and Nelson, 1988a, 1988b, 1989; Pasdar et al., 1991; Mattey and Garrod, 1986b). These studies demonstrated that DP and desmosomal cadherins exist in diffuse cytosolic pools in low calcium conditions. After addition of calcium, DP and Dsg appeared at cell–cell contacts as soon as 15 and 60 min, respectively (Pasdar and Nelson, 1988b; Pasdar et al., 1991). The differences in timing for clustering of these two proteins suggested that junctional desmosomes do not arise from preassembled complexes in the cytoplasm, but rather, assemble at the plasma membrane. Interestingly, some studies in A431 cells and HaCaT keratinocytes that were continuously passaged in low-calcium conditions for several months or even years demonstrated the presence of cytoplasmic complexes positive for both Dsg and DP (Duden and Franke, 1988; Demlehner et al., 1995). These findings suggest that desmosomes can potentially assemble without junctional cell–cell contact and can also internalize as “half-desmosome” complexes. Similarly, in Madin-Darby canine kidney (MDCK) cells switched from normal calcium levels to low calcium, desmosomes underwent splitting and the two halves of the desmosome were internalized within 15 min (Mattey and Garrod, 1986a). These “calcium switch” assays continue to be a valuable tool in desmosome assembly and turnover studies.

**Figure 3.**
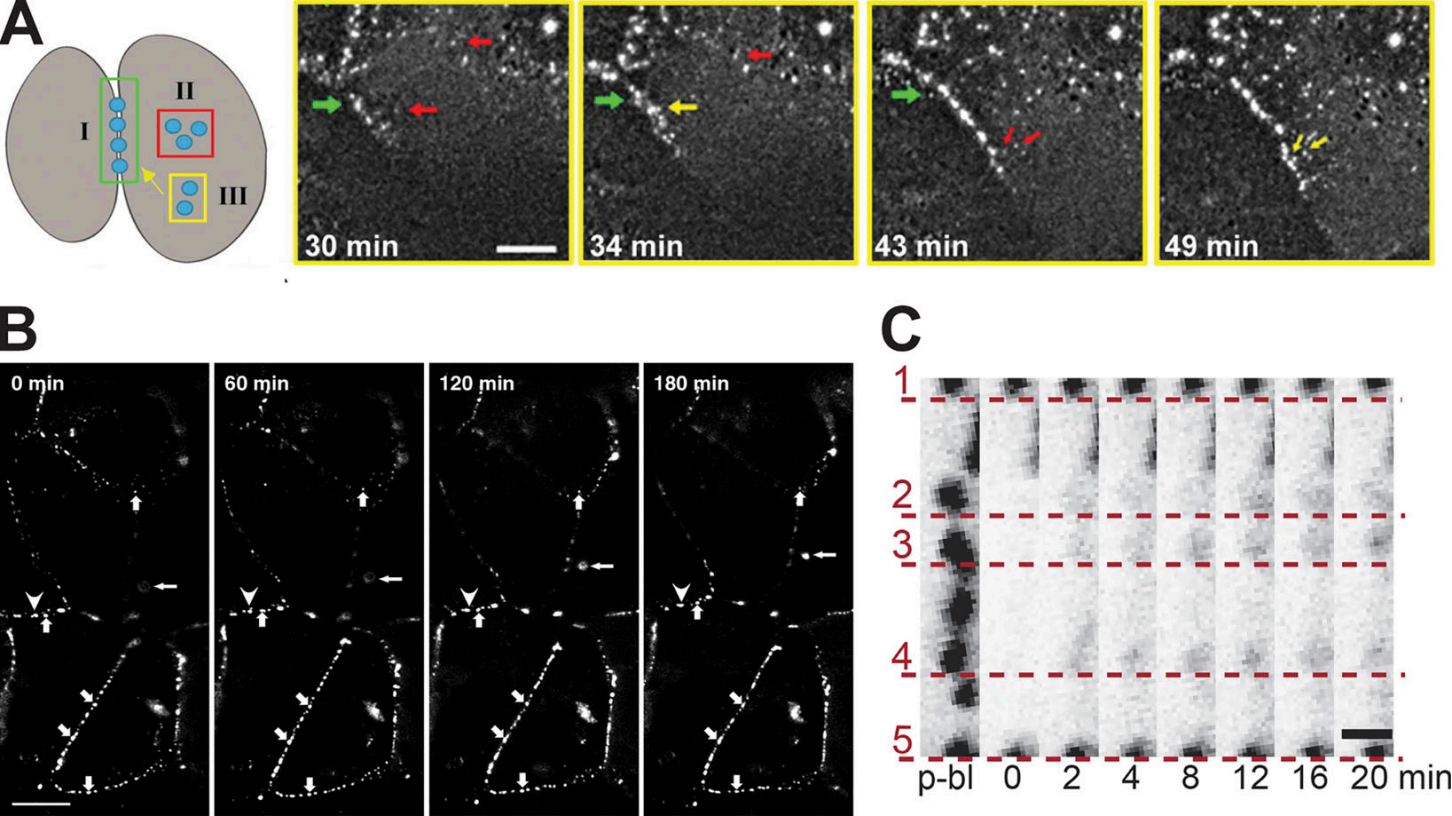
**Visualizing desmosome dynamics in living cells. (A)** Time-lapse recording of the three phases of DP (DP-GFP) assembly in epithelial cell culture after scratch wound (*t* = 0 min). Arrows point to DP puncta and are color-coded by the phase shown in the illustration to the left. Scale bar = 20 µm. Reproduced/adapted from Godsel et al. (2005). **(B)** Sequence of time-lapse images showing Dsc2a (Dsc2a-GFP) puncta fusion (arrowhead) at cell–cell contacts. Arrows mark puncta that remained in the same position throughout the time-lapse. Scale bar = 5 µm. Reproduced/adapted with permission from the *Journal of Cell Science* (Windoffer et al., 2002). **(C)** FRAP demonstrates low mobility of individual Dsg3 (Dsg3-ΔEA-GFP) puncta (numbered 1–5) through time at cell–cell contacts. Scale bar = 1 µm. Reproduced/adapted from Bartle et al. (2020). p-bl: pre-bleach.

The development of genetically encoded fluorescent reporters, such as GFP, revolutionized cell biology and had a profound impact on desmosome research (Tsien, 1998; Chudakov et al., 2010; Chalfie et al., 1994; Heim et al., 1995). Widefield fluorescence microscopy was used to assess DP-GFP dynamics in living human A431 epithelial carcinoma cells (Godsel et al., 2005). Three distinct phases of DP dynamics (Phases I–III) were observed as cells formed new contacts after a scratch wound (Fig. 3 A). In Phase I, DP puncta appeared along the newly forming cell border within 3–10 min. Similar Phase I assembly of DP-GFP has also been observed in a low-to-high calcium switch model of desmosome formation (Bharathan et al., 2023). Furthermore, GFP-tagged Dsc2a and PKP3 also exhibited Phase I behavior after scratch wounding in an oral squamous cell carcinoma cell line (Roberts et al., 2011). Thus, Phase I of desmosome formation is characterized by clustering of desmosomal components at nascent contacts. In Phase II, new DP-GFP particles lacking desmosomal cadherins form in the cytoplasm ∼15 min after cell–cell contact. In Phase III, these newly formed particles are translocated to the cell border. The Green laboratory demonstrated that movement of these DP-GFP particles toward the cell contact was dependent on both keratin IFs and actin but independent of microtubules (Godsel et al., 2005). In contrast, Dsg2- and Dsc2-enriched cytoplasmic vesicles traffic separately to cell–cell contacts on microtubules using kinesin-1 and kinesin-2, respectively (Nekrasova et al., 2011). Moch and colleagues further delineated the sequence of events during desmosome formation in living HaCaT keratinocytes. They concluded that Dsg2 clusters first, followed by the appearance of DP puncta, and subsequent Dsc2 incorporation (Moch et al., 2020). Collectively, these studies suggest that different desmosomal subcomplexes form separately in different regions of the cell. The mechanisms by which these proteins are directed to assemble at spatially constrained regions of the plasma membrane for desmosome maturation remain poorly understood.

Desmosomes at a steady state also exhibit dynamic rearrangement and exchange, although far less than precursor complexes that form transiently during assembly. Windoffer and colleagues showed that Dsc2a-GFP puncta at cell contacts of hepatocellular carcinoma-derived PLC cells were remarkably immobile structures, with little change in shape or fluorescence intensity over durations as long as 3 h (Fig. 3 B) (Windoffer et al., 2002). Occasionally, these Dsc2a-GFP puncta exhibited lateral fusion, which occurred over durations of 30 min to 2 h. The rate of puncta fusion appears to be cell type or desmosome protein specific. For instance, Dsg2-GFP and GFP-PG puncta at A431 cell–cell contacts display more frequent fusion events and undergo lateral fusion within 2–8 min (Gloushankova et al., 2003). Similarly, DP-GFP puncta display lateral fusion in under 3 min in A431 cells (Bharathan et al., 2023). Puncta fusion results in the formation of larger desmosomes from two or more smaller “parents,” which suggests that fusion may maintain desmosome number and/or size, and consequently, cell adhesion. The differences in fusion rates between cell types might result in differential impacts on cell–cell adhesion strength. Further, lateral fusion of desmosome proteins could serve distinct functions under steady-state and dynamic conditions, but these hypotheses have yet to be tested. Lastly, cortical actomyosin tension at edges of cell colonies has also been shown to regulate turnover of Dsg2 and DP-I, but not Dsc2, in HaCaT keratinocytes and MDCK cells (Moch et al., 2022). This finding suggests that increases in tension at the wound edge transduce as-of-yet-unknown signals to augment desmosome formation. Although not discussed here, PKCα (protein kinase C α), epidermal growth factor receptor, and ADAM (a disintegrin and metalloprotease) signaling, as well as Src kinase, glycogen synthase kinase 3, and protein arginine methyltransferase 1 have also been shown to regulate desmosome assembly dynamics during homeostasis and disease states (reviewed in Green et al., 2019; Spindler and Waschke, 2018; Nekrasova and Green, 2013; Müller et al., 2021).

While desmosomes initially form calcium-dependent structures, prolonged periods in confluent culture or inhibition of PKCα causes them to switch into a calcium-independent adhesion state called “hyper-adhesion” (Watt et al., 1984; Wallis et al., 2000; Garrod and Kimura, 2008; Kimura et al., 2007; Garrod et al., 2005). Fluorescence recovery after photobleaching (FRAP) in combination with live-cell microscopy has been used to study protein exchange in these states in cell culture. In calcium-dependent desmosomes of HaCaT keratinocytes, around 20% of desmosomal cadherins (Dsg2 and Dsg3) and plaque proteins (DP and PG) exchange in and out of the junctional complex (Fig. 3 C) (Bartle et al., 2020). This finding further illustrates that desmosomes are not wholly immobile structures. In the hyper-adhesive state however, protein exchange is virtually eliminated, suggesting that proteins are “locked” into a position that maintains adhesion even in the absence of extracellular calcium. Desmosomes in mouse epidermis become hyper-adhesive between embryonic day 12 (E12) and E14 (Kimura et al., 2012), and FRAP experiments demonstrated the stability of desmosomes in mouse epidermis similar to hyper-adhesive desmosomes formed in cell cultures (Foote et al., 2013). In contrast to desmosomes, adherens junctions do not enter a calcium-independent state and the mobile fractions of E-cadherin and the catenins are higher than those of desmosomal proteins (Yamada et al., 2005; Drees et al., 2005; Bartle et al., 2020). Interestingly, the desmosomal plaque protein PKP2a is relatively more mobile than DP, Dsg2, Dsc2, and PG in both calcium-dependent and hyper-adhesive desmosomes (Fülle et al., 2021), suggesting that plakophilin mobility may serve unique assembly or signaling functions.

A key function of desmosomes is to resist mechanical stress. Imaging approaches have again been used to assess forces on desmosomes in both epithelial cells and cardiomyocytes. The development of Förster resonance energy transfer (FRET)–based tension sensors has facilitated the measurement of these forces across desmosomes in living cells. In contrast to adherens junctions (Borghi et al., 2012; Conway et al., 2013), desmosomes appear to be under very little tension in resting MDCK and A431 epithelial cells. However, in MDCK cells, DPI and DPII tension sensors reported increased tension on DP when external mechanical stresses were applied to the cells (Price et al., 2018). Similarly, a Dsg2 tension sensor showed that it was under increased tension in contracting cardiomyocytes (Baddam et al., 2018). The development of a molecular optomechanics approach demonstrated that rapid decoupling of the DP–keratin linkage under mechanical stress disrupts cell–cell adhesion (Sadhanasatish et al., 2023). Collectively, these studies suggest that the desmosome–keratin linkage buffers mechanical stresses that overload actin-based adhesion systems.

In summary, live-cell imaging has provided a view of desmosome dynamics that was inconceivable during early studies of desmosome morphology. In parallel to advances in live-cell imaging, new structural and EM approaches have advanced our understanding of the molecular organization responsible for the paracrystalline desmosome structures first observed by early microscopists, as outlined below.

## Unraveling the molecular architecture of desmosomes

Similar to the emergence of EM in the 1950s and 1960s, the 2000s ushered in a “resolution revolution” in light and electron microscopy (Hong et al., 2023; Jacquemet et al., 2020; Liu et al., 2022). Advances in super-resolution fluorescence microscopy and cryo-electron tomography (cryo-ET) led to new models of protein architecture within the desmosome adhesive core and cytoplasmic plaque. These studies, in conjunction with high-resolution protein structures, are defining, for the first time, how the desmosome puzzle is arranged to yield a mechanically resilient adhesive membrane domain. Perhaps not surprisingly, data from multiple imaging modalities suggests that desmosome architecture is not fixed, but is instead flexible, adopting various arrangements, orientations, and conformations.

## Super-resolution reveals spatial architecture

**Figure 4.**
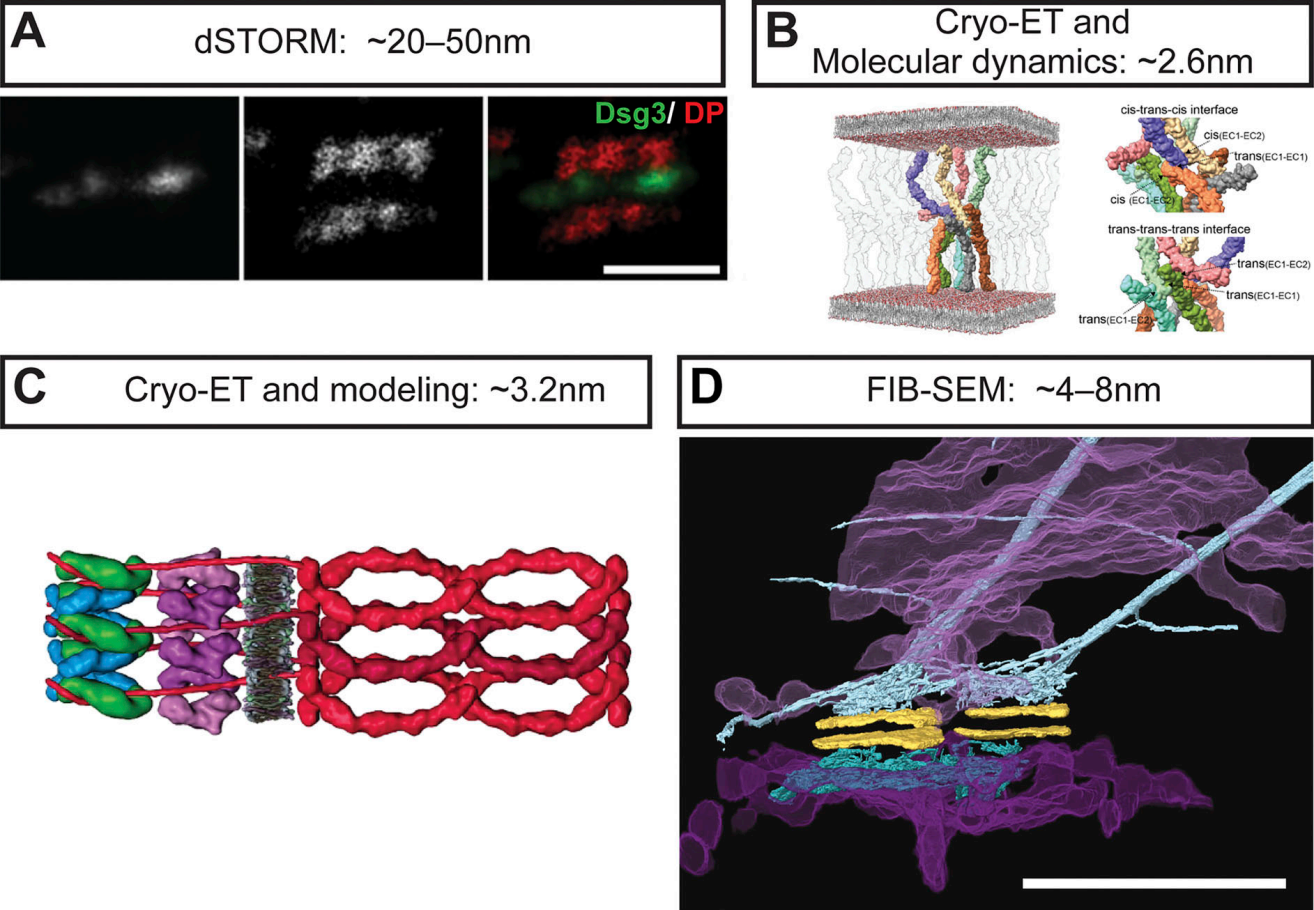
**High-resolution views of the desmosome. (A)** Super-resolution dSTORM imaging provides high-resolution positional information of desmosomal proteins at a cell–cell contact (Dsg3 N-terminus in green and DP C-terminus in red). Scale bar = 500 nm. Reproduced/adapted with permission from the *Journal of Cell Science* (Stahley et al., 2016). **(B)** Cryo-ET and molecular dynamics simulations reveal the cis- and trans-binding interfaces of the Dsg2:Dsc2 heterodimer (Sikora et al., 2020). The cis-trans-cis and trans-trans-trans types of interactions between cadherin EC1-EC2 interfaces are shown on the right. Colors distinguish individual cadherin molecules. Reproduced with permission from Dr. Achilleas Frangakis (licensed under CC BY-NC-ND 4.0). **(C)** An artistic rendering of the desmosome extracellular interface (red—Dsg and Dsc) and outer dense plaque proteins (purple—PKP, green—DP, blue—PG) (Al-Amoudi et al., 2011). This drawing combines information from cryo-ET data in Al-Amoudi et al. (2007), (2011). Reproduced with permission from *Proceedings of the National Academy of Sciences*. **(D)** FIB-SEM and 3-D reconstructions unveil the organization of desmosomes (yellow), keratin filaments (teal), and ER tubules (magenta) in epithelial cell monolayers at nanometer-level isotropic resolution (Bharathan et al., 2023). Scale bar = 1 µm. Resolutions achieved in these studies are stated above each image.

A seminal immunogold EM paper by North and colleagues generated an architectural map of desmosomes from bovine nasal epidermis (North et al., 1999). This map revealed the most likely localization of PG, PKP1, and the N- and C-termini of DP within the desmosomal IDP and ODP. Super-resolution single-molecule localization microscopy approaches, including direct stochastic optical reconstruction microscopy (dSTORM), offer an order of magnitude improvement in spatial resolution over conventional fluorescence microscopy. Stahley and colleagues generated a dSTORM map of desmosome architecture in normal human keratinocytes and human epidermis in good agreement with the North immunogold EM study (Fig. 4 A) (Stahley et al., 2016). Architectural differences in the localization of the DP C-terminus, but not the DP N-terminus, were noted in hyper-adhesive compared to calcium-dependent desmosomes in both cells and tissue. These data suggest that the DP head domain maintains its position in the plaque under various conditions, while the DP C-terminus localizes closer to the plasma membrane in the hyper-adhesive state. A similar change was seen in desmosome assembly where the DP C-terminus was initially more extended into the cytosol and moved closer to the plasma membrane with junction maturation (Beggs et al., 2022). Further studies using super-resolution optical imaging approaches are likely to reveal additional architectural details defining functional states of desmosomes that are not yet appreciated.

## ET reveals protein molecular structure and organization within the desmosome

Early EM imaging suggested a repeating density, possibly resulting from protein organization in the intermembrane space (Rayns et al., 1969). ET was employed with the hopes of elucidating the molecular details of the trans interactions occurring at the desmosome adhesive interface. ET of neonatal mouse epidermis revealed “tangled knots” of desmosomal cadherins (He et al., 2003). Following this study by the Stokes group, Al-Amoudi and colleagues performed cryo-ET on vitreous sections from human skin (Al-Amoudi et al., 2007) and found a regular quasi-periodic pattern of cadherins in the adhesive interface. The two studies had technical differences in sample type, preparation, and implementation of averaging approaches. However, both studies revealed the desmosomal cadherin extracellular domains are not crystalline in arrangement and the exact “order” of the cadherin domains decreases as you move toward the plasma membrane. Both studies used the molecular structure of *Xenopus* C-cadherin to fit sub-tomogram maps, as desmosomal cadherin structures had not yet been solved. Harrison and colleagues solved the crystal structures of full-length ectodomains of human Dsg2, Dsg3, and Dsc1 obtaining resolutions between 2.9 and 3.1 Å (Harrison et al., 2016). They further revealed that heterophilic Dsg:Dsc binding occurred through a strand swap mechanism in trans, strengthened by conserved oppositely charged residues in their respective EC1 (extracellular cadherin 1) domains. More recently, Sikora and colleagues used cryo-ET to image the adhesive interface of mouse liver desmosomes, achieving ∼26 Å resolution, limited by the inherent cadherin flexibility identified by the earlier studies (Sikora et al., 2020). Following fitting and molecular dynamics simulations of Dsg2:Dsc2 heterodimer structures, they proposed a “truss-like” arrangement of cadherins that elegantly reconciled the two previously reported cadherin arrangements (Fig. 4 B). The regularity of the IDP was also characterized by cryo-ET of vitreous sections of human epidermis using the crystal structure of the α-catenin/β-catenin complex to model the DP/PG complex (Fig. 4 C) (Al-Amoudi et al., 2011). Sub-tomogram averaging and fitting of these structures revealed that PG and DP are arranged in alternating patterns, matching the periodicity of the cadherin extracellular interface (Al-Amoudi et al., 2007).

## Polarization microscopy provides insight into cadherin order

An alternate approach to investigate the cadherin interface comes from fluorescence polarization microscopy (FPM). FPM reports the overall angular organization of a tagged protein domain within a diffraction-limited spot. Dsg3 harboring an in-frame enhanced GFP substituted for EC5 was used to measure the order of the most membrane-proximal extracellular domain of Dsg3. The results indicated that Dsg3 is ordered in both calcium-dependent and hyper-adhesive desmosomes in living HaCaT keratinocytes, while this order is lost when calcium is removed regardless of adhesive state (Bartle et al., 2017, 2020). EC5 domain order was confirmed in Dsg2, Dsg3, Dsc2a, and Dsc2b in A431 cells (Dean and Mattheyses, 2022). These data cannot be interpreted as a perfect crystalline-type order, but rather an overall orientational preference that can accommodate deviations. Through modeling, these data support the antiparallel model of cadherin trans binding put forward by the Frangakis laboratory (Sikora et al., 2020). In the antiparallel model, cadherin EC domains are arranged in rows, each rotated 180° relative to the adjacent row on the same cell with the overall pattern rotated by 90° on the opposing cell. Although Dsg2 is recruited early in desmosome assembly, its order was found to be acquired gradually in a calcium switch experiment in A431 cells (Dean et al., 2024). Because of the higher throughput nature of FPM compared with approaches such as cryo-ET, it can more easily be applied to screen desmosome populations and quantify changes in cadherin architecture during dynamic processes in development, wound healing, and various disease states.

## Volume EM unveils a 3-D desmosome and organelle association

While previous studies refined our understanding of the desmosome as a unique plasma membrane domain, highly resolved 3-D views of desmosomes that include a broader cellular and tissue context were lacking. Newly emerging volume EM approaches provide both 3-D information yielded by cryo-ET and larger fields of view achievable with conventional EM. One such approach called focused ion beam scanning EM (FIB-SEM) was first pioneered for use in biological samples by Graham Knott and colleagues (Knott et al., 2008), with improvements in continuous acquisition developed by C. Shan Xu, Harald Hess, and others (Xu and Hess, 2011; Xu et al., 2017). Volume EM workflows involve cutting samples into sufficiently thin sections, capturing 2-D electron micrographs of the underlying sample, and aligning serially captured images into a 3-D “z-stack.” Various modalities of volume EM differ in the EM technique (TEM versus SEM) and the method of sample cutting (Peddie et al., 2022). Light microscopy can also be incorporated into these systems, allowing researchers to answer more complex questions. This correlative light and EM approach enables localizing a fluorescently labeled protein in the corresponding EM image, providing additional biological information in 3-D. Desired sample depth, voxel resolution, and the target feature are all factors that also need to be considered. For instance, in serial block-face scanning EM (SBF-SEM), samples are cut with a microtome, providing 20–50-nm axial resolution (Leighton, 1981; Goggin et al., 2020; Leighton and Kuzirian, 1987; Denk and Horstmann, 2004). With FIB-SEM, samples are typically “milled” with a gallium ion beam, allowing for near-isotropic axial resolutions of <4 nm (Kremer et al., 2015; Bushby et al., 2011; Young et al., 1993). Specific structures in each view are identified and then segmented using manual and deep learning–based approaches. The development of training models using published datasets has facilitated efficient automated segmentation of volume EM data. The segmented images are then used to generate 3-D reconstructions that yield high-resolution volumetric views of organelles and structures of interest (Peddie et al., 2022; Hoffman et al., 2020; Heinrich et al., 2021).

One of the first volume EM studies of desmosomes was performed in the intercalated disc of murine heart ventricles (Vanslembrouck et al., 2018). The intercalated disc is a specialized region at the interface of adjacent cardiomyocytes that contains desmosomes, as well as adherens and gap junctions (McNutt and Fawcett, 1969; Fawcett and McNutt, 1969; Franke et al., 2006, 2007; Borrmann et al., 2006; Vermij et al., 2017). Reconstruction of SBF-SEM and FIB-SEM datasets showed that desmosomes displayed a variety of shapes, sizes, and localization patterns within the intercalated disc (Vanslembrouck et al., 2018, 2020). A recent FIB-SEM study made use of high-pressure freezing to cryo-fix A431 epithelial cells (Bharathan et al., 2023) to preserve near-native cellular ultrastructure (Studer et al., 2008, 2014; Hoffman et al., 2020). The desmosome ODP appeared to have a flat, disc-shaped ultrastructure, consistent with early studies using conventional TEM.

In addition to deciphering the 3-D desmosome ultrastructure, Bharathan and colleagues also revealed an association between desmosomes and the endoplasmic reticulum (ER) (Fig. 4 D) (Bharathan et al., 2023), an organelle first identified by Keith Porter and colleagues in the 1940s as an “intracellular lace-like cytoplasmic network” (Porter et al., 1945). ER tubules were found to make contact with both the desmosome ODP and keratin filaments in A431 cells. ER tubules also exhibited a mirror image–like arrangement on either side of a desmosome, mimicking the organization of keratin filaments. This arrangement was observed in A431 cells, immortalized HaCaT keratinocytes, primary human epidermal keratinocytes, and rat gut and skin. Review of electron micrographs in published literature also reveals ER tubules proximal to desmosomes in human basal keratinocytes, mesothelial cells of *Rana pipiens* (northern leopard frog), and intestinal epithelial cells of *Xenopus laevis* (African clawed frog) (Komura and Watanabe, 1975; Hama, 1960; Hull and Staehelin, 1979). It is possible that recruitment of ER to cell junctions might facilitate localized calcium signaling or maintenance of desmosome lipid raft domains through non-vesicular lipid transfer. A serial EM study showed extensive ER–plasma membrane contacts at intercellular junctions of hepatocytes, being most enriched near tight junctions, but also near desmosomes (Chung et al., 2022). Remarkably, a TEM study conducted in mammalian hepatocytes reported on mitochondria being arranged in mirror images on either side of a desmosome junction, referring to this association as a “des-mite” (Miller et al., 1985). These studies suggest that desmosomes and other cell–cell junctions might serve as organizers of cellular organelles. Such contacts could serve to integrate organelle function with cell–cell adhesion and communication in ways that have not been appreciated.

## Conclusions and outlook

**Figure 5.**
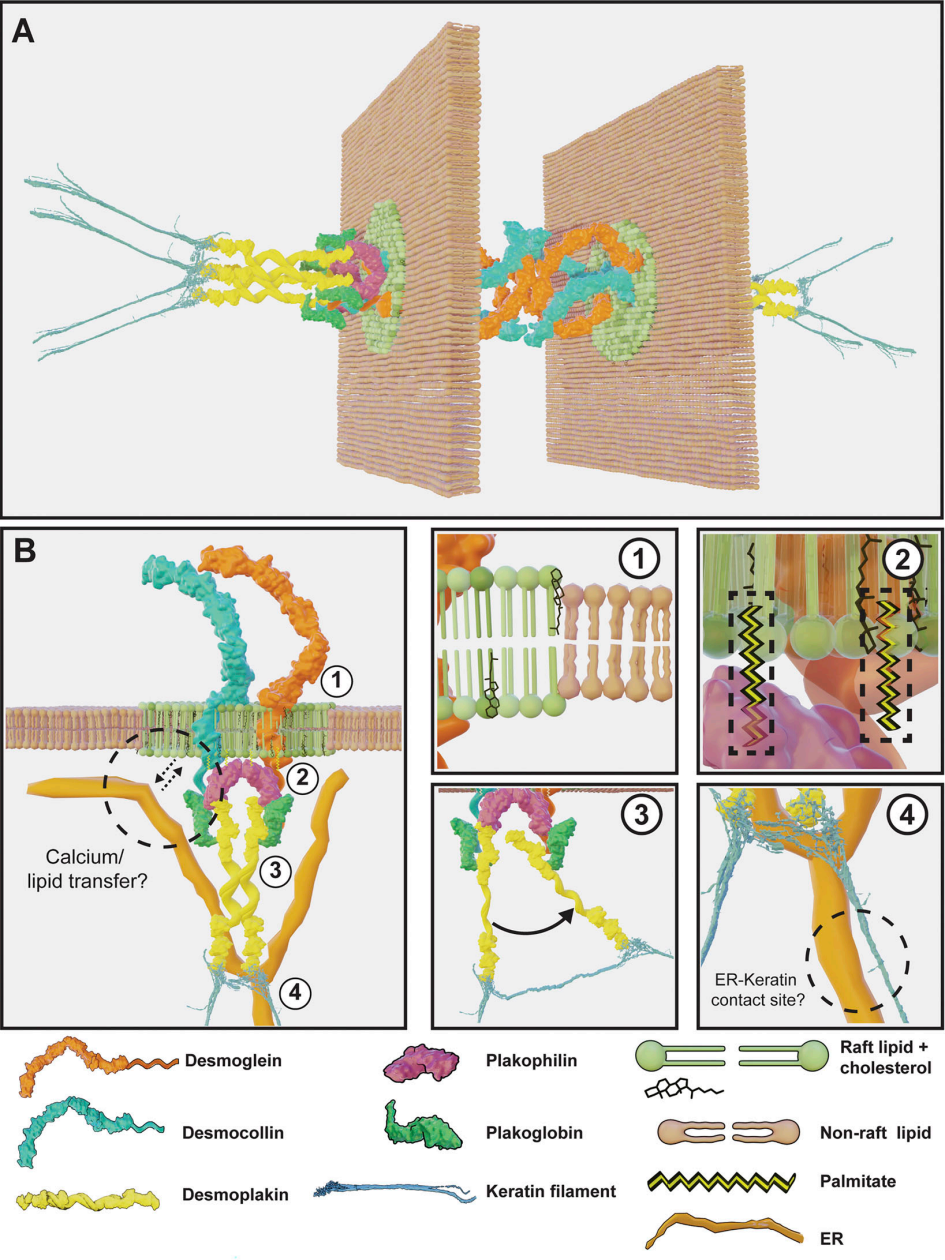
**An updated view of the desmosome cell–cell junction. (A)** Model of a full desmosome across a cell–cell junction. **(B)** Model of the desmosome revealing component protein interactions and lipid microenvironment. The ER (orange tubules) is proximal to the plaque and might regulate localization in calcium and/or lipid transfer functions. Labels 1–4 reveal finer details: (1) Desmosomes are located in thicker lipid raft domains (green), which contain saturated lipids, sphingolipids, and planar cholesterol (black), and are adjacent to relatively thinner non-raft domains (pink), which comprise unsaturated lipid species. (2) Cysteine residues of Dsg2 and PKP2/3 are palmitoylated (yellow palmitate moieties highlighted in dashed black boxes). (3) The C-terminus of DP (yellow) is localized closer to the membrane in mature/hyper-adhesive desmosomes. (4) Keratin filaments (blue) and ER tubules (orange) form close contacts through yet-unidentified linker proteins. The relative sizes of proteins depicted in illustrations are not proportional to actual sizes. Some domain–domain interactions are still unknown.

The evolution of imaging technologies has advanced our understanding of desmosome dynamics and function and provided detailed insights into its structural organization (Fig. 5). Some unique challenges in desmosome imaging remain, however. It has become clear that there is no single “typical” desmosome. Heterogeneity in protein composition, post-translational modifications, dynamics, and architecture represent challenges in data interpretation. For example, optical super-resolution imaging of DP in a population of desmosomes results in a range of possible protein architectures (Stahley et al., 2016; Beggs et al., 2022). As super-resolution imaging advances evolve to achieve higher throughput, so will our understanding of what underlies these variations and their functional implications. Cryo-ET is similarly impacted by the composition and state of the imaged desmosomes. A future frontier in desmosome imaging will require integration of imaging modalities across scales to reveal new insights into protein organization. These approaches will need to incorporate computational tools that can account for protein flexibility and conformational changes intrinsic to desmosome composition and structure.

In addition, biochemical approaches to desmosome biology also remain a challenge due to the detergent insolubility of this complex. The harsh conditions required to solubilize the desmosome–keratin complex, such as high concentrations of chaotropic agents like urea, disrupt native protein conformations and interactions (Pasdar and Nelson, 1988a, 1988b; Nekrasova and Green, 2013). The advent of biotin ligase-based approaches, such as BioID and TurboID, has helped overcome these issues (Branon et al., 2018; Roux et al., 2012). Several studies have used BioID to elucidate desmosomal protein binding partners (Badu-Nkansah and Lechler, 2020; Hegazy et al., 2022; Fülle et al., 2024). These proximity mapping studies continue to advance our understanding of how the interactome of adhesive junctions changes in different adhesion states (Fülle et al., 2024) and at different times and cellular locations (Shafraz et al., 2023). It will also be important to assess the proteome of keratin filaments under these different conditions, especially considering their integral role within the desmosome. Signals might be transduced through the keratin network to intracellular structures including mitochondria and the nucleus (Alvarado and Coulombe, 2014; Wilhelmsen et al., 2005; Lee et al., 2012; Steen et al., 2020; Nishizawa et al., 2005; Duan et al., 2009; Vetter et al., 2020; Kumar et al., 2015), thereby influencing cell adhesion, metabolism, and gene expression.

Novel roles for the desmosome and its constituent proteins are also emerging. For instance, cultured human cardiomyocytes and mouse cardiac muscle lacking PKP2 displayed defective nuclear envelope integrity and increased oxidative stress, leading to DNA damage (Pérez-Hernández et al., 2022). Dsg1 loss in keratinocytes has also been shown to regulate proliferation of adjacent melanocytes in melanomas, which can further repress keratinocyte Dsg1 through paracrine signaling, promoting a feed-forward cycle (Baron et al., 2023, *Preprint*; Burks et al., 2023; Arnette et al., 2020). In addition, loss of adipocyte-specific PKP2 expression was observed in patients with obesity, which was linked to premature adipocyte senescence leading to adipose tissue dysfunction (Lluch et al., 2023). These recent studies highlight the yet unexplored functions of desmosomes and the varied pathomechanisms through which they contribute to disease.

The role of lipid–protein interactions in desmosome organization is also fertile ground for additional imaging and biochemical interrogation. Several early studies of bovine snout desmosome preparations reported high levels of cholesterol and sphingomyelin relative to other lipids (Drochmans et al., 1978; Skerrow and Matoltsy, 1974a). More recent studies using classical lipid raft preparations revealed a striking association of desmosomal proteins with lipid raft membrane domains, perhaps through palmitoylation of membrane-proximal cysteine residues (Fig. 5) (Brennan et al., 2012; Roberts et al., 2014; Resnik et al., 2011; Nava et al., 2007; Lingwood and Simons, 2007; Stahley et al., 2014). In addition, mutations in desmosomal cadherins that abrogate lipid raft association lead to human skin diseases (Lewis et al., 2019; Zimmer et al., 2022). In contrast to desmosomal proteins, most adherens junction components fail to show affinity for raft domains, suggesting that raft association is a key mechanism for desmosome assembly that is distinct from adherens junction formation (reviewed in Zimmer and Kowalczyk, 2020). The recent discovery that ER tubules associate with desmosomes suggests that non-vesicular lipid transfer functions of the ER might facilitate the nucleation and maintenance of lipid domains that facilitate desmosome assembly (Bharathan et al., 2023). Similarly, local modifications of the plasma membrane bilayer by ER lipid transfer mechanisms could aid in the segregation of desmosomes from adherens junctions into specialized membrane domains (reviewed in Zimmer and Kowalczyk, 2024).

In summary, desmosomes are highly resilient structures and exhibit remarkable similarity in ultrastructure across species. A multitude of imaging and biochemical approaches have begun to reveal the fluid nature of desmosomes during the establishment of hyper-adhesion, embryonic development, responses to mechanical stressors, and disease states. These innovations will continue to transform our understanding of desmosome structure and function using principles of a scientific approach first pioneered by the founders of cell biology nearly a century ago.
